# *“The keeping is the problem”*: A qualitative study of IRB-member perspectives in Botswana on the collection, use, and storage of human biological samples for research

**DOI:** 10.1186/s12910-015-0047-3

**Published:** 2015-08-19

**Authors:** Francis Barchi, Keikantse Matlhagela, Nicola Jones, Poloko M. Kebaabetswe, Jon F. Merz

**Affiliations:** Institute for Health, Health Care Policy, and Aging Research Rutgers, The State University of New Jersey, 112 Paterson Street, New Brunswick, NJ 08901-1293 USA; Faculty of Medicine, University of Botswana, Plot 4775, Notwane Road, Gaborone, Botswana; Botswana-UPenn Partnership, 244G - Room 103, University of Botswana Main Campus, Gaborone, Botswana; Department of Medical Ethics and Health Policy, Perelman School of Medicine, University of Pennsylvania, 3401 Market Street, Suite 320, Philadelphia, PA 19104-3319 USA

## Abstract

**Background:**

Concurrent with efforts to establish national and regional biorepositories in Africa is widespread endorsement of ethics committees as stewards of the interests of individual donors and their communities. To date, ethics training programs for IRB members in Botswana have focused on ethical principles and international guidelines rather than on the ethical dimensions of specific medical technologies and research methodologies. Little is known about the knowledge and concerns of current and prospective IRB members in Botswana with respect to export, reuse, storage, and benefit-sharing of biospecimens.

**Methods:**

This qualitative study examined perspectives of IRB members in Botswana about the collection and use of biospecimens in research. Forty-one IRB members representing five committees in Botswana participated in discussions groups in March 2013. Transcriptions of audiotapes and field notes were analyzed to identify issues of concern that might be alleviated through education and capacity-building, and areas that required ongoing discussion or additional regulatory guidance.

**Results:**

Areas of concern included lack of understanding among patients and providers about the use of biospecimens in clinical care and research; reuse of biospecimens, particularly issues of consent, ownership and decision-making; export of specimens and loss of control over reuse and potential benefits; and felt need for regulatory guidance and IRB-member training. Local belief systems about bodily integrity and strong national identity in the construct of benefits may be at odds with initiatives that involve foreign biorepositories or consider such collections to be global public goods.

**Conclusion:**

Education is needed to strengthen IRB-member capacity to review and monitor protocols calling for the collection and use of biospecimens, guided by clear national policy on priority-setting, partnerships, review, and oversight. Engagement with local stakeholders is needed to harmonize fundamentally different ways of understanding the human body and community identity with the aims of contemporary biomedicine.

## Background

Advances in pharmacogenetics and genomics research and their potential to yield public health benefits have contributed to the growing use of human biological specimens (HBS) in research and the establishment, mainly in developed countries, of large-scale biorepositories. Samples from African populations have been included in several public genome reference collections, among them the International HapMap Project, The 1000 Genomes Project, and the Malaria Genomic Epidemiology Network [1–3]. In the past decade, several African biorepositories have been established, including The Gambia National DNA Bank, the African Institute of Biomedical Science and Technology (Zimbabwe) and the biobank at the University of Kwazulu-Natal’s African Centre for Health and Population Studies in South Africa [4–6].

Recently, international efforts to establish a number of regional pan-African biorepositories have gained momentum, largely under the aegis of the Human Heredity and Health in Africa Initiative (H3Africa) [7]. Four pilot regional biobanks are in development- two in South Africa and one each in Nigeria and Uganda. The H3Africa Initiative centers on a ‘hub-and-spoke’ model, with biobanks serving as regional biorepositories of specimens collected as part of research initiatives situated in universities and centers across Africa [8]. It is anticipated that all H3Africa research projects will begin to send a portion or all of their samples to one of these biobanks starting in 2014 [9].

Biobanking introduces highly complex ethical challenges, particularly in Africa where limited access toresearch ethics education and familiarity with drug/treatment development may make it difficult for research participants to understand the risks and the potential benefits associated with genetic research and where regulatory guidance on collection, use, export, ownership and storage of biospecimens is scant [10–12]. Ethics reviewers may lack sufficient understanding about these technologies or have limited access to accurate information on which to base their decisions. “Northern” conceptualizations of self, human origins, risks and potential benefits have shaped approaches to consent, specimen collection, ownership and information sharing — approaches that may be at odds with local meaning-making and therefore of limited value in determining what constitutes ethical practice in these communities [13].

Recent work by Staunton and Moodley identified five studies in the literature that have examined community perspectives on the storage and reuse of biospecimens in countries in Sub-Saharan Africa [12]. Less research has been done to date to gauge the knowledge and perspectives of local ethics committee members who have an essential role to play in protecting stakeholder interests. IRB members may require more information about the science and ethical use of biospecimens in research as well as better policy guidance at a national level if they are to strike the right balance between ensuring safeguards for subjects and their communities on the one hand and facilitating research with potential public health benefits on the other. As efforts to establish biobanks across the African continent expand, due consideration must be given to the extent to which ethics committees have the knowledge to serve as ethical gatekeepers, particularly in communities where local understanding of biomedicine may be rudimentary or at odds with cultural meanings traditionally ascribed to biological phenomena.

Botswana has attracted global attention over the past three decades as a hub for behavioral, epidemiological, and clinical research. A number of foreign academic research universities and NGOs have established study centers in Botswana, including Baylor International Pediatric AIDS, Botswana Harvard AIDS Institute Partnership, otswana-UPenn Partnership, BOTUSA Project, and I-TECH Botswana [14–18]. The University of Botswana opened the country’s first medical school and school of public health in 2009 and has a commitment to advance research and graduate studies as part of its current strategic plan [19]. A number of studies examining genetic attributes of particular sub-populations in Botswana have already begun [20, 21] and the creation of a biorepository at the University of Botswana with the eventual public release of data for future research activities is planned as part of the H3Africa initiative [22].

The Botswana Anthropological Research Act of 1967 provides the basis for research regulation [23]. In 1984, a Health Research Unit (HRU) was created within the Ministry of Health (MoH) and a Health Research and Development Committee (HRDC) established to review, approve, and oversee all health-related research. An Institutional Review Board (IRB) has been in operation at the University of Botswana since 2004; it is responsible for the review and approval of all undergraduate and masters-level research permits and intramural faculty research. It is also tasked with the preliminary review and referral of all health-related PhD and international research to the HRDC for final approval. IRBs have also been in operation for some time at large referral hospitals in Gaborone, Francistown, and Maun. In 2010, the MoH launched a multi-year endeavor to create a country-wide system of local ethics committees to meet the growing demand for ethics review and the expansion of research activities to small communities outside the capital city. Frameworks now exist in a number of the district hospitals for ‘local’ IRBs to provide feedback to the HRDC on the feasibility of projects proposed for their facilities, although most lack infrastructure and research expertise (Kasule M 2014, personal communication). New ethics committees have also been established in various tertiary academic institutions throughout the country, but these are limited in their scope of authority to the review of research proposals by students at the masters level and below. Reviews conducted by this emergent ‘system’ of IRBs are submitted as recommendations to the HRDC, which operates as the national review body for Botswana and remains responsible for final review and monitoring of all health research in the country.

The HRDC is guided by principles articulated in the Declaration of Helsinki, and the Council for International Organizations of Medical Sciences’ International Ethical Guidelines for Biomedical Research involving Human Subjects [24, 25]. Until recently, regulatory guidance on HBS existed only for clinical trials relating to drug applications [26]. In 2011, the Ministry of Health issued Standard Operating Procedures intended to guide the structure and operation of the HRDC and the review of research protocols involving human subjects [27]. A National Health Research Bill remains under review at the parliamentary level.

To date, ethics training programs for IRB members in Botswana have focused on ethical principles and international guidelines rather than on ethical dimensions of specific medical technologies and research methodologies. Little is known about the knowledge, concerns, and training needs of IRB members in Botswana with respect to the use of biospecimens in biomedical research.

## Methods

Members of the HRDC and all IRBs in Botswana at the time of the study were invited by letter with follow-up phone and email reminders to participate in one of several focus groups to be held at various locations in Botswana in March 2013. A total of 41 IRB members drawn from five different committees participated, a sample representing a majority of those members serving on committees tasked with review of protocols involving HBS. Sessions were designed to elicit IRB-member perceptions of common attitudes and beliefs of Batswana (the people of Botswana) towards the collection and use of HBS for research and to gauge member knowledge and attitudes about these issues. Additionally, the research team was interested in IRB-member awareness of existing national regulation regarding the collection and use of HBS, their confidence in its adequacy to guide review, and their perceived need for additional training.

Focus groups took place in Gaborone (two groups of 17 and six participants, respectively) and Francistown (one group of 10 participants), both urban centers in which biomedical research activity has to date been concentrated. One discussion group (eight participants) took place in Mahalapye, a major town to the northeast of Gaborone where the new District Hospital has recently appointed its first IRB. Approximately 70 % of the participants were female. All had received some training in research ethics, but none had received training specific to biospecimens, genetic or genome-based research, or bio-repositories. A number of the members from the HRDC and the University of Botswana were researchers and health professionals familiar with biomedical science. The remaining three committees represented were relatively new, tasked with ‘local’ review of protocols that will be situated in their hospitals and with evaluating their suitability given hospital capacity and local cultural norms and practices. Members of these committees tend to have received less training in ethics compared to their urban counterparts and are less familiar with health research in general. Ethical issues concerning the collection and use of HBS has not been a topic of focus in ethics training programs to date.

Each focus group discussion was guided by a study script. Study questions were chosen with the intention to stimulate a free-flowing conversation in English (the official language of Botswana) to explore IRB members’ own views on the collection and use of biospecimens, as well as what they perceived to be the views of local communities.

Four central questions guided each discussion group:

1. From your perspective, how do the peoples of Botswana feel about the collection of biospecimens?
2. From your perspective, how do the peoples of Botswana feel about biospecimens being kept or stored after the original research has been completed?
3. As an IRB reviewer or someone interested in ethics, what are your greatest concerns about the storage and reuse of biospecimens?
4. Should there be any constraints or conditions placed on the future use of stored biospecimens after the original research is over?

All participants were briefed about the study, given opportunity to ask questions, and were asked for their written consent prior to the start of each session. Focus groups lasted approximately ninety minutes and were audiotaped. Extensive handwritten notes, audio transcripts and observations by the project team over the course of the project formed the basis for categorizing participant responses and identifying themes. The aim was to identify issues of greatest concern to reviewers in Botswana, aspects of those concerns that might be alleviated through education and capacity-building, and areas that might be addressed through ongoing discussion between stakeholders and the international research community or the development of national research guidelines. To minimize the likelihood of misinterpretation, members of the study team solicited feedback on transcriptions of particular passages to confirm or reconsider their understanding of discussant views.

An informal training session about genetic and genomic research was offered at the end of each discussion group. These sessions, intended as a capacity-building effort, were not audiotaped nor were discussions that took place during them considered study data.

The project was reviewed and approved prior to its implementation by the Institutional Review Boards at Rutgers University, the University of Botswana and the University of Pennsylvania, as well as the Health Research and Development Committee at the Botswana Ministry of Health.

For the purposes of the study, a number of words whose conceptual meanings did not directly translate into English were left in *Setswana*, the recognized local language of Botswana. *Batswana* is the collective noun used to describe the people of Botswana. *Go rupa* and *bogwera*, once terms in Setswana (the local language of Botswana) used to describe traditional initiation ceremonies for adolescent boys, is now used by the Botswana MoH to refer to medical male circumcision. *Muti* is the Setswana term used to refer to objects of witchcraft. In describing the ethics system in Botswana, the study used terms commonly used in that country. Ethics research committees in Botswana are referred to as ‘IRBs’ and its members as ‘IRB members’. The IRB at the Ministry of Health is called the Health Research and Development Committee (HRDC).

## Results

### Cultural traditions, norms and beliefs

A significant amount of time in each focus group was devoted to topics having to do with belief systems and cultural norms that were perceived to influence how the collection of biospecimens would be viewed by individuals and communities in Botswana. All four groups referenced Botswana’s current national safe male-circumcision (SMC) campaign to illustrate how cultural meanings can affect local understanding of a medical procedure. Until the mid-20th century when medical missionaries actively discouraged the practice in Botswana, circumcision was part of a month-long rite-of-passage for adolescent boys that taught traditions, values, and ways in which men should relate to women [28]. Meanings that were once attached to circumcision remain, reinforced by the campaign’s use of the Setswana term for the traditional initiation, *go rupa* or *bogwera*, as the term for the medical procedure itself.

*“When* [the campaign] *started,* [health workers] *took their foreskins and just threw them away. Local people were concerned because this is not a tissue. This has meaning. There was a rumor that the foreskins were being sold to traditional doctors. They started thinking that maybe this circumcision is not what they thought it would be.”*

Study participants also observed many Batswana are concerned that foreskins removed as part of this campaign are being sold for other people’s use. Many people, particularly those with less education and those from remote areas of the country, still believe in witchcraft and fear that body parts, including blood and samples that are collected by health workers, will be stolen or sold to make powerful magic (*muti*) that can harm or bring benefit to others.

Discussions of religious beliefs were common to all four groups. Belief that “God has his own way of healing” was seen as discouraging patient utilization of medical services in general, and by extension, of experimental efforts to treat and cure disease. As one discussant characterized a common local sentiment:

*“Why should I allow a medical person to collect a sample from me? If I am sick, for example, if I have cancer; God has his way of healing cancer. Why would I just allow a person who gets his wisdom from God to go and test it? Nature will take its course; it will heal in time if one believes in God.”*

The cultural significance of bodily integrity was seen to stem from the belief that one must return to one’s Maker ‘whole’ and from the importance placed on being able to account for one’s ‘parts’ at the moment of death. One IRB member, describing the case of a young Motswana woman who had had a mastectomy while living outside of Botswana, characterized local fears about loss of bodily integrity in this manner:

*“If these [stored] specimens are out there somewhere in the developed world – far out there- just as I am about to die, can these specimens come back because I want to go to my grave whole? Not to leave part of it over there, overseas? How do these specimens come back? I will not have a peaceful death because I am worried that I am facing death without all my specimens. A breast is somewhere.”*

### Lack of trust

At odds with what IRB members described as Batswana’s ‘tendency to trust’ was the consensus across groups that there was a general lack of trust in research and, by extension, in researchers themselves. Foreign researchers were viewed with greater distrust than local researchers:

*“If you are a foreign team coming from outside and going to the rural areas, there may be suspicion. Even if you are from Botswana and you come with a team – let’s say you are coming from Gaborone to a rural area- they will always suspect that you are going to take something and that you are doing it for something. But if you were from a foreign country, I think it will be even worse.”*

One group discussed at length what it perceived to be the general erosion of trust in clinicians as a direct result of medical research activity in Botswana. What was once trust in the medical doctor to care for the people’s needs has now been replaced by skepticism as to the underlying motives of the health care team. Referring to the current SMC campaign as an illustration of the problem, one IRB member who was a physician offered:

*“I think that* [early on] *they saw the* [actual] *process of circumcision as a medical component – as separate from the rest of the circumcision initiation. They never asked what we were going to do. I think they were confident that we were doing exactly what we said we were going to do… Since research arrived in the country, most people are wary of research, suddenly these issues take on a different complexion. These tests are often not used for the person’s own care. So they don’t know who will see the results or how* [specimens] *will be used.”*

### Misunderstanding of the role of biospecimens in clinical and research settings

Considerable discussion took place in all sessions about the general lack of knowledge in the population about biomedicine, both in clinical and research settings. Participants observed that Batswana on the whole associate the giving of a specimen with clinical care and perceive that in doing so they are contributing to their own well-being. Sick patients come for treatment; physicians draw blood or take a urine sample which goes to the ‘lab’ either for the doctor to use in diagnosing the cause of illness or as an ingredient for a treatment that the nurse or doctor prepares for them in the form of a pill, an injection, or a salve. The patient returns home and in due course feels better. The scenario suggests that often patients associate specimens with treatment, that they believe that their specimens are “used up” in the preparation of that treatment (effectively returning one’s specimen in another form), and that the giving of specimens results in personal benefit. To date, most biomedical research and specimen collection in Botswana has taken place in hospital settings or in association with patient care. Research subjects give specimens because they expect a cure.

*“You know, most Batswana, they expect to get results after you’ve collected a specimen, But now with research, if you take a specimen, you are not going to give any results. Is that right?* [Researchers] *go to families and collect blood specimens. And the results are not revealed to the people. They were advised to go to the hospital to get tested to know their HIV status. So you know from their perspective, they feel cheated. They sometimes feel cheated when it is put that way.”*

Some patients believe that the collection of certain types of specimens, especially spinal fluid, actually worsens health and may hasten death in sick patients. Spinal taps, invariably performed in very sick patients, were reported to be broadly perceived as deadly. In instances of amputation or extraction, it was not uncommon for patients to ask that the limb or tooth be returned to them for ritual disposal.

Several IRB members observed that most Batswana were unaware that samples collected as part of clinical care were generally kept by the medical facility. As one IRB member who was medical staff commented:

*“I think mostly they don’t know that they are being kept. If they knew…they will think that persons are using their specimens to do something else which they didn’t agree to. Mostly they don’t know. They think that their specimen was taken and was used.”*

### Reuse of biospecimens

Questions regarding reuse elicited broadly diverse responses from participants on issues relating to consent, privacy and confidentiality, criteria for reuse, competing research interests, and benefits. A number of IRB members offered that Batswana are likely to be very concerned over specimens being re-used for purposes for which they have not given explicit consent. Several participants felt that a blanket consent for reuse obtained at the time of collection might be acceptable, although they were concerned that research subjects would not fully appreciate the risks and potential benefits associated with such a choice. There was particular concern voiced about the legitimacy of consents obtained from Botswana’s most vulnerable populations, such as the San people of the Kalahari, who may not have fully understood the information on which they were asked to base their decisions.

*“If they are going to take research samples and store them for a long time and then use them for studies that are not disclosed to the individuals, then those persons do not have autonomy over what is going to be don. The researcher needs to at least say something like ‘I am going to use this in the future for HIV research’, and then if the researcher were to [want to] use it for some other purposes, it would not be permitted without permission… I do not think there should be blanket consent, particularly for research that relates specifically to them* [the participants]*.”*

Consent for each new use was the predominant preference among discussants, although there were mixed opinions about the source and nature of such consent. A number of IRB members felt that even when research subjects could give meaningful consent to reuse at the time of collection, requests for reuse should still be reviewed and approved by an IRB or another ‘local group’. Some participants felt that donors should be contacted and new consents obtained for each use, while others were comfortable with review and approvals for future uses by an individual assigned responsibility for the management and supervision of the specimen storage facility. One group agreed that an IRB could approve future uses but emphasized that the reviewing IRB should be a national body, such as the HRDC, and not a local IRB like itself. Those that felt that authority for approval of future use could be delegated to an IRB or other body also felt it was imperative that a strong national regulatory framework be in place so that ‘rules were established and clear’.

IRB members who felt that all reuse was contingent on re-consent by individuals and families from whom the specimens had been obtained stated that specimens needed to be kept in identifiable form for this purpose. Somewhat at odds with this point of view, they were also part of a majority of participants who expressed the view that it was important that specimens be stored in de-identified form for all reuse as a way to ensure privacy. At the same time, most felt that it was important to be able to link data back in the event of discoveries that might be relevant to their health.

There was a fairly lengthy discussion in all groups about the criteria for reuse. Several participants voiced the opinion that many Batswana would be opposed to reuse if the intended uses were not specified. It was felt that researchers would be seen to be “conniving” if they request permission to re-use specimens for unspecified purposes. Those who thought reuse might be acceptable to research subjects shared a number of different viewpoints about the conditions that would apply, including reuse only within the context of the original study, reuse for future studies within the same disease category, reuse during a fixed period of time, and reuse contingent on approval of donors and/or an IRB.

Several groups raised the topic of priority-setting in the context of reuse; they expressed concerns that international researchers might have aims in mind for future use that did not address local needs in Botswana.

*“It should not just be a blanket, open reuse…I shouldn’t* [get] *to reuse specimens just because today I feel like doing research on something that doesn’t return to the nation or to the community. You shouldn’t just be doing research because you just want to learn whatever is happening. There are national and community interests.”*

### Ownership and storage

Discussions regarding ownership elicited strong opinions from all the IRB members. Some members felt that specimens belong to the person who gave them, and that individual donors should retain control and access to them, even in storage, and should be able to retrieve them if they so choose.

*“If I donate a sample for research as a participant, that sample is mine. That belongs to me…My DNA and everything is there. That belongs to me… I need some control or comfort level with my sample.”*

Several IRB member participants expressed the minority opinion that biospecimens should be the property of the researcher or the research institution to whom they were originally given. Even though several participants suggested that there might be gradual transference of ownership between researcher and the nation, and that specimens may for a time have to be located outside the country, the majority of participants agreed that ultimately the specimens should belong to Botswana. The government, they felt, should be the ultimate gatekeeper.

*“I think that if I get specimens for my research, there should be a point at which they don’t belong to me anymore, when they ought to belong to the community of researchers or to the human community.* [But] *there should also be regulations at different levels. You know, if the specimens are here at some stage, they have to be transferred from me either to the university or to the country and the country will take custody of those specimens for future generations and everybody else.”*

The common belief that specimens are “used up” in an individual’s care was seen by most IRB members as a major obstacle to acceptance by Batswana of HBS reuse and storage. One discussant observed “the keeping is the problem,” conveying in one phrase local concerns that specimens would not be wholly used for a person’s own care, that specimens would be “out there somewhere” at the time of death, and that someone else without the same obligations to self, community, and country would control them.

One group voiced distinctly different views about duration and benefits of storage. This group included the more ‘seasoned’ IRB members from the HRDC and the University of Botswana. These participants introduced some pragmatic considerations not voiced by others.

*“My concern is the fact that once specimens have been collected, at some point, people* [will be] *required to destroy them. It’s such a waste, you know…. To say after three years, ‘okay, destroy them’ doesn’t seem to me to be a good solution. It presumes that we are going to do harm with that specimen* [and that] *we* [the IRB] *are not watching. We just throw away specimens. One hundred years from now, researchers would benefit from specimens if they can keep them that long. But unfortunately we throw them away.”*

IRB-member attitudes towards storage were linked to concerns about access. Many felt that storage of specimens could be of benefit if priority access was given to local researchers wishing to conduct research of direct benefit to Botswana. There was general agreement that a regulatory framework was needed to ensure safeguarding of specimens regardless of duration of storage.

There was widespread consensus among participants that, ultimately, specimens should be kept in Botswana.

*“I have a problem with the specimens going out. Why? Because you never know what they are going to be used for. You have no control over them once they have left the country. I’d rather prefer whoever wants to do that research should have a budget that is enough to bring whatever equipment or facility here and do whatever he wants to do in the country so we can benefit.”*

Participants acknowledged the need to build capacity in-country for storage, safekeeping, and research, with most voicing the view that the international research community should help in this effort.

### Potential benefits and benefit-sharing

Concerns about reuse and storage were generally linked to considerations about how individuals, communities, and the nation could benefit. Complicating such discussions was the conflation of ‘payment’ to research subjects for their time and inconvenience at point of specimen collection with ‘benefit’. Because many IRB members viewed this practice as payment to subjects for the use of their sample or information, it followed that subjects should continue to be paid (in their words ‘benefit’) each time their samples were re-used.

IRB members felt it was important that any benefits derived from the use of stored specimens, including monetary gains, intellectual property rights, or new treatments, should be shared with the individuals who provided the specimen, their communities, and/or the nation as a whole. Most participants recognized that this is already a concern with current single-study use that would become more pronounced when specimens were stored and reused, particularly if storage was located outside of Botswana.

Several participants felt that concerns over benefits were exacerbated by a general lack of feedback to research subjects about study results, particularly in light of widespread confusion about ‘research’ versus ‘clinical care’, from which patients routinely received some benefit, often in the form of a medication, treatment, or a return to health.

*“If they* [research subjects] *are not given results, it affects the future and the chances that others will give specimens. How come you always come to us and never give us feedback?”*

Others remarked on the woeful lack of recognition of the contribution that research subjects make. Seeing such recognition as a form of benefit, it was their opinion that a lack of such acknowledgement had a negative impact on people’s willingness to participate in research.

*“We see nowadays people who have contributed to the good work, contributed to the research in many ways….how do we recognize* [and] *reward the person, from whose specimens we learned something important?....Reward does not need to be monetary… If the people contributed to an achievement in research, they should be recognized and acknowledged. If our grandfathers built a school, we continue to be proud of this school because it wears our grandfathers’ names.”*

### Educational needs and regulatory guidance

Study participants were unanimous in their recognition of the need for more education and training opportunities for IRB members, particularly on issues relating to genetic and genome-based research. They agreed that all committee members must be knowledgeable about issues relating to biomedical research, research ethics and, in particular, the use of biospecimens. A number of participants, particularly those from the newer IRBs, voiced frustration at the lack of information they had on which to base their deliberations and the limited recognition that was given to the amount of time required for review.

*“The official training would help. It would be better than what we are now. We don’t want to be biased. If we tell someone that they have to comply, we need to know… For some* [IRB members] *its core business, but the rest of us are dragged in. If you knew this was ‘certified’ business, you would work more at it.”*

*“We came here to do other things; we are* [co-] *opted to be in the IRB. I don’t even have time to think or read on my own, or to learn what the expectations are. And I have to read so many papers.”*

When asked if they were familiar with any regulatory guidance for research and if so, if they felt it was adequate, most study participants agreed that such guidance existed, but were generally not familiar with it or felt that it was ‘easy to bypass’. All but one IRB member believed that there were existing regulations about biospecimens. Most participants were familiar with the existence of a forthcoming Health Research Law and expressed general anticipation that this law would address existing inconsistencies and concerns with respect to access, ownership, and storage of biospecimens and benefit-sharing.

It was generally felt that regulations would increase the consistency of IRB reviews and would reduce the perception of inconsistent, discretionary decision-making by IRB members.

*“*[A national policy] *would be very important. See, right now, either we don’t know things well, or we can bypass them. If there were clear regulations, we would be able to prevent people from operating differently. Tomorrow I am going to be able to say, ‘This is the law’. A proper legislation, along with penalties and everything. It will apply to everybody. That would be better.”*

## Discussion

A number of the issues raised by study participants echo findings from other studies in developing countries that examined community attitudes and norms towards biomedical research. Similar concerns about the use of human ‘parts’ for sorcery or witchcraft have previously been reported in the academic literature as has the attribution of cultural meaning to parts of the human body [29–33].

Bodily integrity, particularly at death, has been identified as a critical value in various cultural and religious traditions and one with significant ramifications for research [34, 35]. To what extent the meaning given to bodily integrity might cause communities to resist the donation of biospecimens for future research and storage is new to considerations about biobanking in Africa and warrants further study, particularly in the context of genetic and genomic research involving biosamples in Botswana.

Trust has also been identified elsewhere as central to the research subject–researcher relationship and in the willingness of individuals to participate in research [31, 32, 36, 37]. IRB members in Botswana emphasized the absence of trust as a significant problem, attributing this in part to lack of understanding on the part of Batswana and lack of adequate explanation and study feedback on the part of researchers. This failure to engage with communities adequately has been cited previously as a cause for erosion of trust in researchers in developing countries [38]. De Vries and colleagues observed that trust between researchers and research subjects may be further diluted when samples are exported for use by strangers in foreign countries [10].

IRB-member views that Batswana are likely to be resistant to the reuse of their specimens is in sharp contrast with earlier findings from Uganda that 95 % of research subjects interviewed would be comfortable with the reuse of their biospecimens contingent on IRB review [39]. A recent study by Igbe and Adebamowo also found that lay persons in Nigeria, once biobanking was explained to them, felt that storage and reuse was a ‘worthwhile’ activity [40].

Donor consent or local approval for reuse was preferred by the majority of IRB members in this study, a point of view they felt accurately represented how Batswana in general would feel and how they themselves felt as members of the IRB. Most agreed that review and decision-making could be delegated to a knowledgeable IRB or other regulatory body in Botswana. Review and approval by bodies outside the country was generally not considered adequate protection for the interests of the individuals and communities from whom the biospecimens had been collected.

Ownership was a particularly contentious topic in this study, reflecting a consensus that samples ultimately belonged to the people of Botswana and by extension should be under the care and protection of the national government. Similarly, Moodley and colleagues found that most of the perspectives voiced by research subjects in South Africa regarding sample storage, export, reuse, and benefit-sharing ultimately had to do with ownership [32]. Participants in that study, despite discussions about ‘donations’, persisted in referring to ‘my blood’ or ‘my child’s blood’ just as IRB members in this study repeatedly emphasized “my specimen”, “my samples”, or commented “if I donate a sample for research as a participant, that sample is mine”.

The expectation that an individual’s samples will be used up in the course of his or her own care may be viewed by Batswana as a fair exchange — the ‘giving up’ of a specimen in exchange for treatment or return to health — that is absent when they are asked to provide a specimen for the purposes of research. Certainly there is concern among IRB members that societal benefits may be absent if specimens are taken outside the country or if decisions as to their future use do not remain in the hands of the donors themselves or the government of Botswana. IRB members in Botswana discussed benefits both in very basic terms of compensation to research subjects themselves and in broader language suggesting that they view biosamples as a collective resource that should be used primarily for the benefit of the country and all its citizens.

Familiarity with pharmacogenetics and genomic research was limited in IRB members participating in this study. Participants on the two most active IRBs – the national HRDC and the IRB at the University of Botswana – were more comfortable discussing the scientific aspects of research and more likely to consider the potential benefits of genetic- and genomic-based research than their counterparts in the newer and less active IRBs. There was a uniform call from all IRB members for more training in these areas, a need that has been expressed by similar constituencies in other African countries [41–44].

Uniformly absent from the discussions in all groups, regardless of their familiarity with research or their experience as IRB members, were several topics of particular relevance to biomedical research in Botswana. First, the concept of ‘benefit’ was narrowly viewed by IRB members only within the context of individual, community, and national interests of Botswana. Little, if any, consideration was given to the potential benefits that genetic- and genomic-based research using biospecimens from Botswana could offer other peoples in Africa or other populations worldwide sharing common traits, risk-profiles or diseases despite follow-on questions on this issue from the study team. This consideration of benefit only within the geographic boundaries of their country may simply reflect what IRB member participants perceived as the ‘local’ focus of this current study. However, if the discussants’ comments are symptomatic of a broader territorial protectionism, it suggests that there may be some resistance to proposals that call for export of biospecimens to regional or non-African biorepositories located outside of Botswana. Concerns such as these, for example, have been noted previously in South Africa [45]. In particular, it may reflect a negative view of management and reuse of such specimens by foreign investigators without local oversight. Such attitudes could be directly at odds with the ‘common heritage’ approach that underpins many international and regional strategies for biobanking, including the current H3Africa initiatives [7, 46].

Second, despite the near unanimity of IRB members as to the lack of understanding on the part of research subjects in Botswana and the particularly disturbing reports that participants are frequently unaware that they are subjects of research or that their specimens are not exhausted in their own care, the IRB members participating in this study did not evidence any sense of personal responsibility as ‘gatekeepers’ to address such concerns. Despite the national mandate to its IRBs to “ensure that the dignity, rights, safety and well-being of research subjects are protected” [27], and the IRB members’ uniform call for more ethics training, there was little evidence that the IRB members at present saw their boards accountable for the remediation of ethical ‘gaps’ in how studies are actually taking place on the ground. Training requirements for new IRB members, combined with continuing professional development opportunities for current members, can certainly help. The success of such efforts, however, will depend on the existence of clear regulatory guidance as well as adequate investment in research governance and support structures. All IRB members need to be given access to training, time-off from other responsibilities, and recognition for the value of their service if they are to function as effective, meaningful stewards of citizen safety, rights, and dignity in research.

### Limitations of this study

The authors chose to study the perspectives of IRB members in Botswana in order to understand the challenges they face as ethical gatekeepers responsible for the review of increasingly sophisticated research involving the collection, use, and storage of HBS, and to assess the need from by IRB members for specialized training in this domain. To that end, the study provides insights through their eyes only on community knowledge, attitudes, and beliefs about biospecimens. To address this limitation, more studies are needed in Botswana that directly engage local communities in order to appreciate what is likely to be the broad range of understanding and opinion on ethical, legal, and social implications associated with biospecimens, particularly those collected as part of research biorepositories. The need for such discussions have been recently highlighted by the H3Africa Working Group on Ethics [47]. At the same time, opportunities for more in-depth debate among IRB members in Botswana regarding areas of concern to the constituencies they protect would help to tailor both future IRB-member training and public health outreach. Information provided through studies involving other non-Botswana populations as well as international guidance on ethical concerns at the community-level in developing countries offer some help in navigating the terrain but are not sufficient to fully understand local complexities nor identify points of opportunity for education, participation, and collaboration that are most appropriate for Botswana.

## Conclusion

A number of IRB-member concerns identified in this study may well be addressed through education and capacity-building efforts. Greater investment on the part of the Botswana health system as well as the international research community in community-level education could reduce misconceptions about how biospecimens are used, sharpen the distinction in patient understanding between clinical care and medical research, and engender a greater appreciation for the potential benefits afforded their communities by new advances in research. Opportunities for community engagement, both pre- and post-research, should be addressed in all study protocols, with clearly articulated plans for community feedback. Future education and training activities for IRB members can address the current knowledge gap on genetic- and genomic-based research, the risks and benefits of specimen reuse, regional biobanks, shared bioinformatics, and the current regulatory guidance for review of related protocols. Capacity-building programs are needed to develop local expertise in Botswana’s own growing research community and to create the necessary physical and professional infrastructure to manage the safe and ethical collection, utilization, and storage of biospecimens. Once developed and enacted, clear national policy on the export, ownership, and terms of use for biospecimens in research can provide a guide for research priority-setting, partnerships, review, and oversight.

This study also identified some areas that may prove less malleable to change. These include indigenous meanings ascribed to the human body and its parts that seem to be at odds with ‘western’ scientific knowledge and practice. Engagement by the international research community with local stakeholders will be needed to harmonize these fundamentally different ways of understanding the human body and community identity with the aims of contemporary biomedicine.
